# Do online social interactions cultivate social capital? Evidence from a longitudinal study

**DOI:** 10.3389/fpsyg.2022.989137

**Published:** 2022-10-14

**Authors:** Dong Zhou, Yanan Li, Tanin Tirasawasdichai

**Affiliations:** School of Media and Communication, Shanghai Jiao Tong University, Shanghai, China

**Keywords:** online social interactions, trust, charitable donation, a longitudinal study, cross-sectional studies, structural equation modeling

## Abstract

It is widely documented that social capital can benefit individual and social development. However, research on the roles of internet technologies in cultivating social capital has not arrived at a consensus. This article aims to understand the effects of online social interactions (OSIs) on generalized trust and prosocial civic engagement, two essential forms of social capital, with a longitudinal study and structural equation model. Fixed-effect model estimations consistently show that OSIs can effectively increase levels of generalized trust in China. Also, trust in parents is used as an alternative dependent variable to provide a comparative analysis. The mechanisms of these two sources of trust are different, and insignificant effects of OSIs on trust in parents are found to implicitly support the causal link between OSIs and trust in strangers. In this study, we implemented a series of robustness checks, for example, examinations using only the netizens as a sample and cross-sectional methods. Furthermore, we explored the relationship between OSIs and prosocial civic engagement (charitable donation), a behavior form of social capital. The SEM results suggested that charitable behaviors were positively affected by OSIs and generalized trust played a positive mediating role. Additionally, significant positive direct and indirect effects through the generalized trust in OSIs were found on prosocial behaviors.

## Introduction

Social capital is a complex and multidimensional concept encompassing institutions, social networks, and cultural and social value systems (Bhandari and Yasunobu, 2009; Glanville and Bienenstock, 2009). It is widely acknowledged to be beneficial to individual and social development by improving wellbeing, increasing social engagement, lowering crime rates, enhancing productivity, promoting economic growth, and resulting in better community development (Quan-Haase and Wellman, 2004; Welch et al., 2005; Dearmon and Grier, 2009; Van Groezen et al., 2011; Bologna, 2014; Portes and Vickstrom, 2015; Bjørnskov, 2017; Kalyanamitra, 2018; Paarlberg et al., 2018; Boontham, 2019; Wilmot and Dauner, 2019). Thus, it is important for us to understand its causes and effects.

Today, the world faces a crucial and dramatic transition with social and economic challenges related to the advancement of digital technologies. The global internet penetration rate now stands at 59.5%, and around 4.66 billion people worldwide use the internet (Kemp, 2021). As of December 2021, the number of internet users in China has reached 1.01 billion, accounting for 71.6 % of the total population in China. The adoption of internet technology in industry 4.0 has positively impacted productivity and sustainable growth in Small and Medium-sized Enterprises (Haseeb et al., 2019a,b). But, the key question remains: does individual internet usage contribute to social capital accumulation?

Existing literature shows that there are no commonly agreed answers to this question. Information and communication technology used to be viewed as a threat that kept people from civic engagement and compromised social capital [see Bowling Alone by Putnam (2000)]. Empirically, Olken (2009) found that television had crowded out social participation, and Geraci et al. (2022) observed that internet access caused a significant decline in social participation and social capital among the British. In contrast, Huysman and Wulf (2004) argued that the internet could contribute to new forms of interaction and community, followed by interpersonal relationships and social networks. Bauernschuster et al. (2014) stated that there was no evidence supporting the claim that internet access has reduced social capital, and conversely, there even exists significant positive effects of the internet on some particular dimensions of social capital. Liu et al. (2016) provided a thorough meta-analysis of the positive relationship between social networking online and social capital. They argued that social networking sites offer a platform to strengthen existing interpersonal relationships.

Trust, as one important collective asset, promotes relationships and networks and enhances the utility of embedded resources, and vice versa (Lin et al., 2001). Generalized trust, specifically, is an important social capital and is different from trusting known persons, and its relationship with internet use has attracted the attention of many researchers. Mutz (2009) found that online commerce experiences promoted generalized social trust with experimental data. Näsi et al. (2015) discovered that online viewing of negative images and reading hate material were negatively correlated with social trust, and the effect was larger on acquaintances than on generalized trust. Sabatini and Sarracino (2019) found that all forms of trust significantly decreased with participation in online networks in Italy. Zhou et al. (2020) found that internet usage negatively affected political and generalized trust, but not trust in parents, in a 2015 Chinese General Social Survey data. However, Lu et al. (2020) identified a significant positive effect of internet use on trust in governments among netizens in a 2017 Netizen Social Consciousness Survey.

As noted above, different conclusions have been arrived at in different countries and sometimes even in the same country but at different time periods. There are two major concerns in the existing literature. First, internet use is a complex multidimensional issue encompassing different usage functions and qualities. A simple broad measure is insufficient to understand the influences of the internet on social trust. Second, most of the studies simply provide cross-sectional analyses and are not sufficient for causal inferences. This compels the need for panel data studies or more advanced research designs. For example, a longitudinal study serving as a control for individual-level fixed effects can help alleviate individual-level endogenous bias.

Computer-mediated communication theories such as the Hyperpersonal Model suggest that sufficient online social interactions (OSIs) can match or even exceed social networks generated by face-to-face interactions (Walther, 1996). Currently, most social activities have moved to online environments, especially during the pandemic. Social networking sites and social media platforms incredibly facilitate interpersonal exchanges and accelerate interactions between the known and the unknown. Therefore, it is expected that OSIs, particularly, could generate positive effects on trust and prosocial behaviors. This paper examines the effects of OSIs on social capital cultivation with longitudinal data from China. Specifically, we investigate generalized trust toward strangers and charitable behaviors.

## Method

### Data and measures

The dataset we used is drawn from 2014, 2016, and 2018 series of Chinese Family Panel Studies (CFPS). This is a nationally representative biennial panel survey of Chinese residents employing a multi-stage stratified sampling procedure and interviewing individuals from 31 provinces. We focused on the sample of adults born between 1950 and 1990 who were at least aged 24 in 2014, and almost all of the participants had completed their education. Through the unique personal identification numbers, we could track them over time and construct a panel dataset for our empirical study. In all, a total of 16,655 respondents were tracked from 2014 to 2018 and included in our main empirical data.

#### Dependent variables

*Generalized Trust* toward strangers is the main dependent variable followed by Bjørnskov (2007). The related question used is “how much do you trust in strangers?” The respondents answered by selecting a score between 0 to 10, indicating the lowest to the highest level. Supplementary Table A shows that the level of generalized trust increased from 2014 to 2018, but the mean value, 1.985, was relatively low. *Trust in parents* is used to make comparisons. Respondents were also asked directly, “how much do you trust in parents?” and answered by selecting a score between 0 to 10. Unlike trust in strangers, trust in parents was scored at a high level ranging between 9.326 and 9.425.

We then studied how OSIs affect the charitable donation behavior of respondents. Related questions were surveyed only in 2018. Respondents were directly asked whether they had donated or not during the past year, whether they had donated online, and how much they had donated. We constructed three associated dependent variables, *donation, donation amount*, and *online donation*. Based on whether the respondent had donated or not, *Donation* was represented as one or zero, respectively. *Donation amount* was determined by the total amount of the donation behavior and it was taken natural log. *Online donation* was a categorical variable where two represented donating through the internet, one represented offline donation behavior, and zero represented all those who had not donated. We found that about 23.3% of the respondents had made charitable donations.

#### Key explanatory variables

The critical explanatory variable was OSIs, and two measures were used to study it. The first was *whether OSIs* (one, yes; zero, no), and the second was *frequencies of OSIs* measured from zero to six (zero representing never used; one representing once in several months; two representing once a month; three representing two to three times a month; four representing one to two times a week; five representing three to four times a week; and six representing every day). From 2014 to 2018, the proportion of respondents using the internet increased from 26.55 to 48.76%, and correspondingly, the ratio of respondents interacting online increased from 20.9 to 43.7%. The mean value of the frequency of OSIs increased from 1.022 to 2.344 and the proportion of respondents interacting online every day increased from 10.07 to 28.62% between 2014 and 2018.

#### Covariates

A wide range of socio-demographic covariates were considered in the estimations: gender (1 = male, 0 = female), place of residence (0 = urban, 1 = rural), perceived social status (score 1-5 from the lowest to the highest), age (in years), marital status (1 = Married/cohabitating, 0 =single/divorced), retirement status (0 = retired; 1 = labor market), education (0–22 years of schooling), party membership (1 = communists, 0 = no), and self-reported health condition (score 1–5 from poor to healthy). Please see Supplementary Table A for variable statistics by year.

### Analyses

#### Fixed-effect models

Hausman test (χ^2^= 310.96) and Sargan-Hansen test (χ^2^= 269.48) were run for modeling. Both test results supported selecting the individual fixed effect model rather than the random effect. Thus, our main empirical model for the longitudinal study was set up as follows:


(1) $$\begin{array}{l} {\;Trust}_{it}=\lambda_{i}+\beta_{1}OSI_{it}{+\varphi X}_{it}{\;+\;\eta }_{o}{\;+\;\varepsilon }_{it} \end{array}$$

where *o* indicates province o, *i* indicates individual i, and *t* indicates survey year t. The coefficients of the OSIs were expected to be positive. λ_*i*_ is a group of individual indicators serving as a control for individual fixed effects and filtering out contaminations from unobserved personal characteristics, e.g., personality. Unobserved determinants of trust (generalized trust or trust in parents) that are fixed at the provincial level, such as regional living standards, cultures, and customs, were controlled through provincial indicators (η_*o*_). *X*_*it*_ is the vector of covariates presented above. ε_*it*_ is the error term.

#### Cross-sectional analyses

We further investigated the effects of OSIs on prosocial social participation (charitable donation behaviors) with the 2018 survey of the CFPS. That is because only the 2018 survey had donation-related questions. Linear multivariable regression equations were utilized to study the relationships between OSIs and donation, donation amount, and online donation. Finally, we analyzed the association between OSIs and donation behavior and the mediating effect of generalized trust using structural equation modeling (SEM). The structural equation model was conducted using *donation* as the dependent variable, while *generalized trust* and *trust in parents* were entered as mediators. *Whether OSI* was the main predictor. All regressions controlled for covariates listed above. The data analyses were performed in Stata, version 16 and maximum likelihood estimations and bootstrap replications of 1,000 times were used.

## Results

The estimation results of equation (1) are presented in Table 1. *Frequencies of OSIs* were found to generate a significant and positive impact on *generalized trust*. The coefficient was 0.027, significant at the level of 1%. The effect was larger when we used the dummy measure, *Whether OSIs*. Those using the internet for social functions showed a higher level of generalized trust of 0.101, around 5.1% of the mean. The effects were much larger, approximately doubled, when we ran regressions with the subsample of just the netizens (β = 0.056, *p* < 0.01, for *frequencies of OSIs*; β = 0.218, *p* < 0.01, for *whether OSIs*). However, we did not find a significant impact of OSIs on *trust in parents* (0.003 with *frequency of OSIs* as the independent variable; 0.006 for *whether OSIs*).

**Table 1:** The effects of OSIs on generalized trust.

	**Main results**		**Placebo test**		**Subsample of netizens**	
**Dependent variables**	**Generalized trust**		**Trust in parents**		**Generalized trust**	
	**(1)**	**(2)**	**(3)**	**(4)**	**(5)**	**(6)**
Frequencies of OSIs	0.027^***^		0.003		0.056^***^	
	(0.007)		(0.003)		(0.010)	
Whether OSIs		0.101^***^		0.006		0.218^***^
		(0.037)		(0.018)		(0.059)
Constant	1.326^***^	1.341^***^	9.153^***^	9.158^***^	1.638^***^	1.709^***^
	(0.136)	(0.135)	(0.061)	(0.061)	(0.213)	(0.212)
Observations	49,958	49,963	49,960	49,965	16,241	16,245
R-squared	0.563	0.563	0.522	0.522	0.661	0.660

Coefficients of covariates are not reported for brevity.

Robust standard errors are clustered to birth cohort level and are reported within parentheses.

*** represent significance at 1% level; ^**^represent significance at 5% level; ^*^represent significance at 10% level.

Next, we undertook robustness checks using cross-sectional analyses and the results are presented in Table 2. In all the ordinary least square regressions, we similarly developed a control for social status, health conditions, marital status, retirement status, education attainment, urban or rural residential, communism membership, gender, age, age square, provincial, and birth cohort fixed effects. After all the samples were used, wave year fixed effects were added. As presented, all coefficients were consistently positive and significant (β = 0.046, *p* < 0.01, for *frequencies of OSIs*; β = 0.224, *p* < 0.01, for *whether OSIs*). Without providing control for individual fixed effects, the effect sizes were larger compared to results with control for individual fixed effects in Table 1.

**Table 2:** Robustness checks with cross-sectional analyses.

**Cross-sectional analyses**	**Dependent variable: generalized trust**				
**Sample**	**2014–2018**	**2014–2018**	**2014**	**2016**	**2018**
Frequencies of OSIs	0.046^***^		0.040^***^	0.071^***^	0.025^***^
	(0.005)		(0.010)	(0.008)	(0.007)
Whether OSIs		0.224^***^			
		(0.026)			
Constant	−0.418	−0.508	−2.525	−6.233	−10.185
	(0.481)	(0.481)	(6.923)	(6.770)	(7.544)
Observations	49,958	49,963	16,653	16,653	16,652
R-squared	0.058	0.058	0.048	0.069	0.061

Notations are the same as Table 1.

*** represent significance at 1% level; **represent significance at 5% level; *represent significance at 10% level.

The results of our investigation on the effects of OSIs on charitable donation behavior is presented in Table 3. Again, consistent, and robust positive correlations between OSIs and donation behaviors were found. OSIs increased the probability of the person making donations during the past year (β = 2.3%, *p* < 0.01 for *frequencies of OSIs*; β= 11.9%, *P* < 0.01 for *whether OSIs*), enhanced the amount of donation (β= 12%, *p* < 0.01 for *frequencies of OSIs*; β = 61.5%, *p* < 0.01 for *whether OSIs*), and promoted the probability of choosing to donate online (β = 3.9%, *p* < 0.01 for *frequencies of OSIs*; β = 20.3 % *P* < 0.01 for *whether OSIs*).

**Table 3:** Associations between OSIs and prosocial behaviors.

**Dependent variables**	**Donation (0–1)**	**Donation amount**	**Online donation**	**Donation (0–1)**	**Donation amount**	**Online donation**
**Methods**	**OLS**	**OLS**	**OLS**	**OLS**	**OLS**	**OLS**
Frequencies of OSIs	0.023^***^	0.120^***^	0.039^***^			
	(0.00)	(0.00)	(0.00)			
Whether OSIs				0.119^***^	0.615^***^	0.203^***^
				(0.00)	(0.00)	(0.00)
Observations	20,787	20,790	20,790	20,790	20,793	20,793
R-squared	0.121	0.133	0.138	0.119	0.131	0.136

In all analyses, we introduced control for social status, health conditions, marital status, retirement status, education attainment, urban or rural residential, communism membership, gender, age, age square, and provincial and birth cohort fixed effects. Results are available upon required. Robust standard errors are clustered to birth cohort level and are reported within parentheses.

*** represent significance at 1% level; **represent significance at 5% level; *represent significance at 10% level.

**Figure 1:**
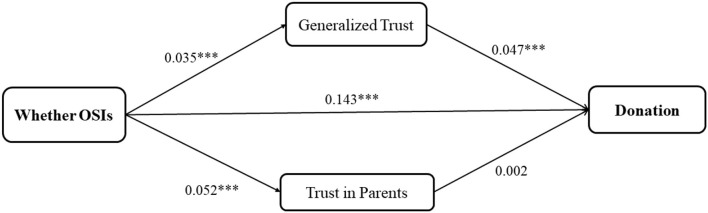
Results of structural equation modeling showing the effect of OSIS on donation and mediating effects of trust. Note: Only important standardized coefficients are reported for brevity. All regressions include the series of covariates as table 3 and maximum likelihood are used to estimate with bootstrap replications of 1000 times. Robust model fit indices: N-20707. = 1.5. degree of freedom 1.CFI 0.999. TLI 0.997, RMSEA 0.0505. SRMR0.001. *p < .1, *p* < .05.*p.001, SRMR is not reported because of missing values.

The SEM results with respect to *donation* are presented in Figure 1. For brevity, only standardized coefficients of interested paths are represented. There was a positive association between OSIs and donation (β = 0.143, *p* < 0.001). Higher levels of trust in strangers and parents were found among those who used the online interaction function (β= 0.035, *p* < 0.001, for *generalized trust*; β=0.052, *p* < 0.001 for *trust in parents*). *Trust in parents* did not generate a significant effect on donations. However, trust in strangers was positively associated with donation (β = 0.047, *p* < 0.001). Thus, OSIs work by increasing *generalized trust* which in turn promotes charitable donation behaviors.

## Discussion and conclusion

Our findings support that frequent online social interactions indeed extend to reality and contribute to social capital in China. First, fixed-effect model estimates and the comparative study show that OSIs generate higher levels of generalized trust toward strangers. Respondents socializing online marginally exhibit higher generalized social trust by 0.101 compared with those who do not, and by 0.22 compared with netizens who do not. Online social interactions can contribute to bridging social networks, psychologically making citizens close, and thus increasing generalized trust. This finding is consistent with the Social Information Process Theory and Hyperpersonal Model that argue effective online communication with sufficient interaction time can enhance social networking and cultivate interpersonal trust (Green and Clark, 2015).

Second, we found that the impact of OSIs on trust in parents was insignificant. It was because the mechanisms of trust in strangers and parents differ. Trust in parents is determined by family interactions. Even though there exists a positive relation between OSIs and trust in parents in the cross-section analyses, the effect disappeared when we ran fixed-effect models. Influenced by the Confucian culture and moral system, the Chinese are traditionally inclined to trust those with whom they have personal relationships (kinship or quasi kinship) rather than strangers, in contrast to the spirit of the “stranger ethic” of modern Western philanthropy (Yang and Zhu, 2020). However, as internet use and OSIs become universal, Chinese citizens are likely to change these traditional attitudes. Gradually, it is expected to enhance levels of generalized trust and contribute to social capital cultivation.

Third, a positive association between OSIs and donation behaviors was found. Trust as a fundamental element of social capital is closely associated with community development such as promoting charitable behaviors (Shah et al., 2001; Wellman et al., 2001; Irwin, 2009; Herzog and Yang, 2017; Kalyanamitra, 2018). For those who are social online, the probability of making a donation is 11.9% higher (particularly, larger for donating online than other offline donation behaviors) and the amount donated is likely to be 61.5% higher. In general, China's performance in philanthropy lags behind western countries. With digital technologies being increasingly adopted, philanthropy in China is set to expand significantly in China.

Lastly, we found that an important pathway in this positive relationship was through increasing levels of generalized trust, but the mediating effect of trust in parents was insignificant. The significant mediating effect of generalized trust exists in the relationship between OSIs and charitable behaviors. Individuals who engage in OSIs more frequently are likely to trust strangers and the enhanced generalized trust further leads them to participate in more prosocial behaviors. Philanthropy and charity have generally worked as a way of social redistribution for alleviating social inequality, improving the lives of the disadvantaged, providing public goods, and enhancing sustainable development. Thus, internet generalization and a higher level of social capital cultivated by OSIs are beneficial to community development.

Our paper not only pioneers exploring the causal link between OSIs and generalized trust, but also innovates by investigating the effect of OSIs on prosocial civic engagement as well as its mechanism. Our results shed light on policy implications: not only expansion in digital technology investment but also special function training programs are needed.

One limitation of our research is that because survey questions change, we did not have information on donation behaviors in 2014 and 2016 and only cross-section analyses were implemented for the relationship between OSIs and donation behaviors. When more panel datasets are available, more causal inferences can be offered through studies with longitudinal methods. Also, diverse forms of social capital and other functions of the internet can be studied in the future.

## Data availability statement

The raw data supporting the conclusions of this article will be made available by the authors, without undue reservation.

## Ethics statement

Ethical review and approval was not required for the study on human participants in accordance with the local legislation and institutional requirements. Written informed consent from the patients/participants or patients/participants legal guardian/next of kin was not required to participate in this study in accordance with the national legislation and the institutional requirements.

## Author contributions

DZ is in charge of planning, designing, and writing the paper. YL organizes the empirical results and collects and reviews the literature. TT takes care of data merging and data analyzing. All authors contributed to the article and approved the submitted version.

## Conflict of interest

The authors declare that the research was conducted in the absence of any commercial or financial relationships that could be construed as a potential conflict of interest.

## Publisher's note

All claims expressed in this article are solely those of the authors and do not necessarily represent those of their affiliated organizations, or those of the publisher, the editors and the reviewers. Any product that may be evaluated in this article, or claim that may be made by its manufacturer, is not guaranteed or endorsed by the publisher.
