# Supplementary material for: Do online social interactions cultivate social capital? Evidence from a longitudinal study
Source: Front Psychol. 2022 Oct 14;13:989137. doi: 10.3389/fpsyg.2022.989137 (PMC9614328; doi:10.3389/fpsyg.2022.989137)
Supplement: Supplementary file 1 [file Data_Sheet_1.docx]

**[Supplementary Materials](https://review.frontiersin.org/Document/DownloadSupplementaryMaterial?articleId=917039&userId=1495728&roleId=17)**

1. **Data Source and Basic Statistics**

The CFPS is conducted by the Institute of Social Science Survey (ISSS), funded by Peking University. The surveys collected data at the individual, family, and community levels and are designed to track changes in Chinese society, economy, demography, education, health, etc. The data were officially launched in 2010, and thus far tracked in 2012, 2014, 2016, 2018, and 2020. The original sample covers 25 provinces (municipalities directly under the central government and autonomous regions) in China with targeted sample size of 16,000 households. It focuses on the economic and non-economic well-being of the Chinese population, and contains rich information on economic activities, educational attainment, family relationships and dynamics, migration, physical and mental health, and so on. We use the 2014, 2016, and 2018 waves. Most of the observations keep the same, but still some changes in interviewees over time. Applying for the personal identification numbers, we can track them over time and construct a panel dataset for our empirical study. Also, the questionnaires vary by year. For example, only the 2018 wave asks respondents about donations.

**Table A：Statistics Descriptions of the Constructed Panel**

| **Year** | **2014** |  | **2016** |  | **2018** |  |
| --- | --- | --- | --- | --- | --- | --- |
| Persons: 1,7175 | **Mean** | **SD** | **Mean** | **SD** | **Mean** | **SD** |
| **Generalized Trust:** 1-10 from lowest level of trust to highest level of trust in strangers | | | | | | |
|  | 1.894 | 2.070 | 1.902 | 2.088 | 2.160 | 2.196 |
| Trust in Parents (0-10) | 9.416 | 1.371 | 9.425 | 1.343 | 9.326 | 1.424 |
| **Frequencies of OSIs:** | 1.022 | 2.085 | 1.783 | 2.597 | 2.344 | 2.750 |
| **Whether OSIs** | 0.209 | 0.407 | 0.335 | 0.472 | 0.437 | 0.496 |
| Social Status: | 2.952 | 0.989 | 2.823 | 1.056 | 3.130 | 1.075 |
| Marital Status: | 0.916 | 0.277 | 0.919 | 0.273 | 0.915 | 0.279 |
| Health Status: | 3.035 | 1.219 | 2.914 | 1.207 | 2.888 | 1.217 |
| Retirement Status: | 0.085 | 0.279 | 0.019 | 0.136 | 0.138 | 0.345 |
| Education Attainment | 7.531 | 4.520 | 7.580 | 4.552 | 7.384 | 4.815 |
| Communism Membership | 0.067 | 0.251 | 0.084 | 0.277 | 0.091 | 0.288 |
| Age | 45.337 | 11.277 | 47.038 | 11.330 | 48.936 | 11.397 |
| Male | 0.480 | 0.500 | 0.480 | 0.500 | 0.480 | 0.500 |
| Urban Residency | 0.455 | 0.498 | 0.475 | 0.499 | 0.493 | 0.500 |

1. **More Empirical Results**

| **Table B. More Results for Relationships between OSIs and Prosocial Civic Engagement** | | | | | | |
| --- | --- | --- | --- | --- | --- | --- |
| **Panel A** |  |  |  |  |  |  |
| **Variables** | **Donation**  **(0-1)** | **Donation**  **(0-1)** | **Online Donation** | **Donation**  **(0-1)** | **Donation**  **(0-1)** | **Online Donation** |
| Methods | Probit | Logit | Probit | Probit | Logit | Probit |
| Frequencies of OSIs | 0.077*** | 0.131*** | 0.082*** |  |  |  |
|  | (0.00) | (0.00) | (0.00) |  |  |  |
| Whether OSIs |  |  |  | 0.413*** | **0.712***** | 0.439*** |
|  |  |  |  | (0.00) | **(0.00)** | (0.00) |
| Observations | 20784 | 20784 | 20790 | 20787 | 20787 | 20790 |
| Pseudo R2 | 0.116 | 0.116 | 0.101 | 0.115 | 0.115 | 0.100 |
| **Panel B** |  |  |  |  |  |  |
| **Variables** | **Donation**  **(0-1)** | **Donation**  **(0-1)** | **Donation Amount** | **Online Donation** | **Online Donation** | **Online Donation** |
|  | (1) | (2) | (1) | (2) | (1) | (2) |
| Generalized Trust | 0.008*** | 0.008*** | 0.045*** | 0.043*** | 0.012*** | 0.011*** |
|  | (0.00) | (0.00) | (0.00) | (0.00) | (0.00) | (0.00) |
| Whether OSIs |  | 0.118*** |  | 0.612*** |  | 0.201*** |
|  |  | (0.00) |  | (0.00) |  | (0.00) |
| Observations | 20744 | 20744 | 20747 | 20747 | 20747 | 20747 |
| R-squared | 0.109 | 0.121 | 0.122 | 0.133 | 0.123 | 0.137 |

Note: In all analyses, we control for social status, health conditions, marital status, retirement status, education attainment, urban or rural residential, communism membership, gender, age, age square, provincial and birth cohort fixed effects. Results are available upon required. Robust standard errors are clustered to birth cohort level and are reported within paratheses.

**Figure 1: Variations of OSIs from 2014 to 2018**


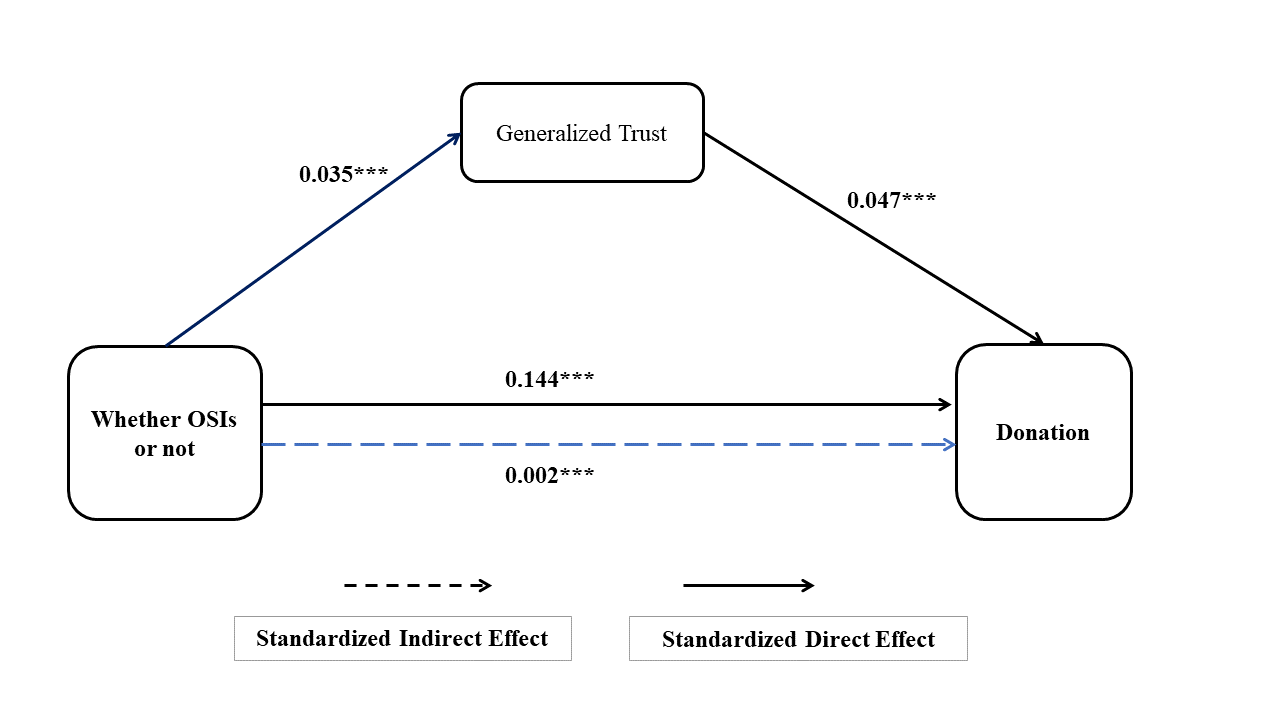


**Figure 2: Standardized Direct and Indirect Effect of OSIs on Donation Obtained from SEM Estimations with 2018 CFPS**
